# Metabolic and RNA sequencing analysis of cauliflower curds with different types of pigmentation

**DOI:** 10.1093/aobpla/plac001

**Published:** 2022-02-05

**Authors:** Daozong Chen, Yingxia Yang, Guobao Niu, Xiaozheng Shan, Xiaoli Zhang, Hanmin Jiang, Lili Liu, Zhenghua Wen, Xianhong Ge, Qiancheng Zhao, Xingwei Yao, Deling Sun

**Affiliations:** 1 College of Life Sciences, Ganzhou Key Laboratory of Greenhouse Vegetable, Gannan Normal University, Ganzhou 341000, China; 2 Tianjin Academy of Agricultural Sciences, The State Key Laboratory of Vegetable Germplasm Innovation, The Tianjin Key Laboratory of Vegetable Genetics and Breeding, Tianjin 300384, China; 3 National Key Laboratory of Crop Genetic Improvement, College of Plant Science and Technology, Huazhong Agricultural University, Wuhan 430070, China; 4 Tianjin Huierjia Seeds Industry Technology Co., Ltd, Tianjin 300392, China

**Keywords:** Anthocyanin, cauliflower, metabolome, transcriptome

## Abstract

Cauliflower (*Brassica oleracea* var. *botrytis*) is a popular vegetable worldwide due to its delicious taste, high nutritional value and anti-cancer properties. Cauliflower normally produces white curds, and natural spontaneous mutations lead to the production of orange, purple or green curds. However, some white cauliflowers show uneven purple pigmentation in their curds, which seriously affects the appearance quality and economic value of this crop. The underlying mechanism is still unclear. In this study, we performed comparative transcriptional and metabolic profiling analysis of light orange, white and purplish cauliflower curds. Metabolite analysis revealed that the pigments conferring purple colouration were delphinin and cyanin. Transcriptome analysis showed that the anthocyanin metabolism-related structural genes *DFR*, *ANS* and *UGT* and the transcription factor genes *PAP2*, *TT8*, *GL3*, *EGL3* and *TTG1* were upregulated in purplish versus white curds. These findings shed light on the formation of purplish curds, which could facilitate the breeding of purely white or red cauliflower.

## Teaser

It is a worldwide problem that cauliflower curds turn purplish at low temperatures, which seriously affects the quality of the product. The molecular mechanism is not clear so far. This study will reveal the causes of purplish curds under low temperature stress for the first time at the genetic level, provide conclusive molecular evidence for the accurate creation and improvement of cauliflower resources and provide a new theoretical approach for solving the problem that curds turn purple under low temperature stress in cauliflower.

## Introduction

Cauliflower (*Brassica oleracea* var. *botrytis*) is one of the most important vegetables worldwide due to its numerous nutritional and health benefits (Manchali *et al.* 2012). *Brassica oleracea* vegetables contain compounds that can reduce the risk of age-related chronic diseases and several types of cancer (Lampe *et al.* 2002; Podsędek *et al.* 2007; Jahangir *et al.* 2009). China has the largest planting area and most output of cauliflower worldwide. In 2018, cauliflower production in China reached 10.26 million tons, accounting for 40.67 % of the world’s total output (FAO 2019).

Cauliflower can produce white curds as well as coloured curds, such as orange, purple and green curds. Although coloured curds have gained increasing interest as functional foods, white cauliflower is the most popular for traditional cooking globally. However, white cauliflower curds can sometimes show slight purple pigmentation due to the biosynthesis of anthocyanins in response to various environmental factors, which dramatically reduces their market value. Therefore, the production of stable white curds under various natural conditions is an important breeding goal of cauliflower.

Anthocyanins are water-soluble pink/red/purple/blue flavonoid pigments that are widely distributed in various plant tissue. These secondary metabolites, which accumulate in different vegetative or reproductive plant organs, attract pollinators and seed carriers (Hatier and Gould 2008; Yuan *et al.* 2013) and enhance plant resistance to biological and abiotic stresses (Gould 2004). Main anthocyanins can be divided into three categories based on the number of hydroxyl groups in the B ring: cyanidin (orange to red), pelargonidin (magenta) and delphinidin (blue to purple). Further modifications including methylation, glycosylation and acylation lead to the production of three main types of anthocyanins, pelargonin, cyanin and dephinin in nature. These modifications, the binding of anthocyanins with different metal ions, and intracellular pH all affect the final colour of anthocyanins (Glover and Martin 2012; Ren *et al.* 2015).

The anthocyanin biosynthetic pathway and related genes have been well characterized in *Arabidopsis thaliana*, maize (*Zea mays*), petunia (*Petunia hybrida*), snapdragon (*Antirrhinum majus*) and other plant species (Broun 2005; Dixon *et al.* 2005; Koes *et al.* 2005; Grotewold 2006). Phenylalanine is the first precursor for the biosynthesis of anthocyanins and other flavonoids. The biosynthetic pathway of anthocyanins from phenylalanine to anthocyanin is generally divided into three steps: 4-coumarate-CoA biosynthesis, flavonoid biosynthesis and anthocyanin biosynthesis. The genes involved in both flavonoid and anthocyanin biosynthesis, including the genes encoding chalcone synthase (*CHS*), chalcone isomerase (*CHI*) and flavanone 3-hydroxylase (*F3H*), are known as early biosynthesis genes (EBGs). By contrast, the genes involved only in anthocyanin biosynthesis, including genes encoding dihydroflavonol reductase (*DFR*), leucoanthocyanidin dioxygenase/anthocyanidin synthase (*LDOX/ANS*) and UDP-glucose: flavonoid 3-*O*-glucosyltransferase (*UFGT*), are called late biosynthesis genes (LBGs). Anthocyanin biosynthesis is mainly regulated at the transcriptional level via genes encoding key enzymes, especially LBGs. These genes are regulated by a class of transcription factor complexes called MBW (MYB–bHLH–WD40) complexes. In *Arabidopsis*, various proteins participate in the formation of these complexes, including GL3, EGL3 and TT8 from the bHLH family, PAP1, PAP2, MYB113 and MYB114 from the R2R3-MYB family, and the WD repeat protein TTG1 (Shi and Xie 2014).

In many plants, the specific accumulation of anthocyanins in different tissues is primarily due to the activation of R2R3-MYB transcription factors (Chiu *et al.* 2010; Shi and Xie 2010, 2011; Butelli *et al.* 2012; Chiu and Li 2012). Gene expression analysis suggested that the accumulation of anthocyanins in vegetative tissues of *B. oleracea* is caused by the active expression of R2R3-MYB transcription factors (Yuan *et al.* 2009; Zhang *et al.* 2014b, 2015). Genetic mapping and gene function analysis showed that in kale, kohlrabi and cabbage, the formation of purple traits is specifically due to the high expression levels of *BoMYB2*. In purple types of these vegetables, transposon insertion or sequence variation is present in the promoter region of this gene (Chiu *et al.* 2010; Chiu and Li 2012; Yan *et al.* 2019), resulting in a significant increase in its expression.

During the process of cauliflower breeding, we obtained two lines: ZF-206 and FQ-36. Whereas ZF-206 always produces light orange curds, some curds produced by FQ-36 are white, but this line is extremely sensitive to temperature and readily accumulates purplish pigments on the curd surface in winter. In the current study, we compared the transcriptomes and metabolomes of the light orange curds of ZF-206 with the purplish curds of FQ-36 during different stages of development. We determined that the formation of purplish curds results from the accumulation of cyanin and delphinin, which might occur due to the upregulation *EGL3* and *PAP2*.

## Materials and Methods

### Plant materials and growth conditions

Seeds of the pure cauliflower lines ZF-206 (with light orange curds) and FQ-36 (with purplish curds) were sown in the Wuqing experimental field of Tianjin Academy of Agricultural Sciences, China on 1 August 2017, and the young plants were transferred to the greenhouse on 20 September 2017. Samples (with three biological repeats) were collected from light orange curds of ZF-206 and white, light purplish and fully purple curds of FQ-36 during different developmental stages. All samples were collected and immediately frozen in liquid nitrogen for RNA and anthocyanin extraction.

### RNA extraction, reverse-transcription PCR, and qRT-PCR analysis

RNA for library construction of each sample included three replicates per treatment and genotype, giving a total of 12 samples. Total RNA was extracted from each sample using an Eastep Super Total RNA Extract Kit (Promega, Shanghai, China) supplemented with RNase-free DNaseI to remove contaminating DNA according to the manufacturer’s instructions. The quality of the purified RNA was evaluated on a 2 % agarose gel and quantified using a NanoDrop™ spectrophotometer (Thermo Fisher Scientific, Inc.). RNA purity was checked using a NanoPhotometer® spectrophotometer (Implen, CA, USA), and RNA concentration was measured using a Qubit® RNA Assay Kit in a Qubit® 2.0 Flurometer (Life Technologies, CA, USA). RNA integrity was assessed using an RNA Nano 6000 Assay Kit from the Bioanalyzer 2100 system (Agilent Technologies, CA, USA). First-strand cDNA was synthesized using a RevertAid First-Strand cDNA Synthesis Kit (Thermo, USA, https://www.thermofisher.com/cn/zh/home.html). The cDNA was amplified on a CFX96TM Real-time PCR Detection System (Bio-Rad, Germany, http://www.bio-rad.com/). Three genes were selected for quantitative real-time PCR (qRT-PCR) confirmation of the expression level revealed by RNA-seq analysis. The specific quantitative primers for different genes and their transcripts were designed using Primer 5.0. qRT-PCR assays with three technical repetitions of each sample were performed using a Luna Universal qPCR Master Mix (Biolabs, USA) on a Bio-Rad CFX96 Real-Time Detection System (Bio-Rad, Germany, http://www.bio-rad.com/). The Bnaactin3 gene was used as an internal control for data normalization, and quantitative variation in the different replicates was calculated using the delta-delta threshold cycle relative quantification method as described previously (Chen *et al.* 2020b).

### Library construction and sequencing

RNA for library construction was prepared using 3 µg of RNA per sample. Sequencing libraries were generated using a NEBNext Ultra RNA Library Prep Kit for Illumina (NEB, USA) following the manufacturer’s recommendations, and index codes were added to each sample. Briefly, mRNA was purified from total RNA using poly-T oligo-attached magnetic beads. Fragmentation was carried out using divalent cations at elevated temperature in NEBNext First-Strand Synthesis Reaction Buffer (5×). First-strand cDNA was synthesized using random hexamer primer and M-MuLV Reverse Transcriptase (RNase H−). Second-strand cDNA synthesis was then performed using DNA Polymerase I and RNase H. The remaining overhangs were converted into blunt ends using exonuclease/polymerase. Following adenylation of the 3′ ends of the DNA fragments, NEBNext Adaptor with hairpin loop structure was ligated to the fragments to prepare them for hybridization.

To preferentially select cDNA fragments 250–300 bp in length, the library fragments were purified using the AMPure XP system (Beckman Coulter, Beverly, MA, USA). The size-selected cDNAs containing adaptor sequences were combined with 3 µL USER Enzyme (NEB, USA) and incubated at 37 °C for 15 min, followed by 5 min at 95 °C. PCR was then performed using Phusion High-Fidelity DNA polymerase, Universal PCR primers, and Index (X) Primer. The PCR products were purified (AMPure XP system) and library quality was assessed on the Agilent Bioanalyzer 2100 system. Clustering of the index-coded samples was performed on a cBot Cluster Generation System using a TruSeq PE Cluster Kit v3-cBot-HS (Illumina) according to the manufacturer’s instructions. After clusters were generated, the library preparations were sequenced on the Illumina HiSeq platform, and 125 bp/150 bp paired-end reads were generated. All four samples were subjected to transcriptome sequencing with three biological repeats.

### RNA-seq data analysis

Low-quality reads were removed from the raw reads using Cutadapt (Martin 2011) and Trimmomatic (Bolger *et al.* 2014) software. Clean reads were mapped to the *B. oleracea* var. TO1000 genome sequence (Parkin *et al.* 2014) using TopHat2 (Kim *et al.* 2013). The FPKM of each gene was calculated based on the length of the gene and the read counts mapped to this gene. FPKM, the expected number of Fragments Per Kilobase of transcript sequence per Million base pairs sequenced, considers the effect of sequencing depth and gene length for the reads count at the same time. Gene expression levels were calculated using Cufflinks (Trapnell *et al.* 2012).

### Differential gene expression analysis

The read counts of each gene were calculated using the htseq-count function in htseq software (Anders *et al.* 2015). The R package DEseq2 (1.16.1) was used to identify the differentially expressed genes (DEGs) between curds of different colours based on the following criteria: *P*adj < 0.01 and log_2_FoldChange > 2.

### Identification and expression analysis of anthocyanin-related genes

Protein sequences from *A. thaliana* were download from https://www.arabidopsis.org/. Homologous genes were identified by BLAST analysis and confirmed based on similarity scores. Gene collinearity analysis was performed using MCScanX software (Wang *et al.* 2012). MEGA7 software was used for evolutionary analysis (Kumar *et al.* 2016). TBtools software was used to draw a heat map of the FPKM values of anthocyanin-related genes (Chen *et al.* 2020a).

### Sample preparation and anthocyanin extraction

Freeze-dried curd samples were crushed in a mixer mill (MM 400, Retsch) with a zirconia bead for 1.5 min at 30 Hz. Each 100 mg sample of powdered tissue was extracted overnight at 4 °C in 1.0 mL of an aqueous solution of 85 % methanol: formic acid (V_methanol_:V_ddwater_:V_formic acid_ = 8:15:0.5). Following centrifugation at 10 000*g* for 10 min, the extracts were absorbed (CNWBOND Carbon-GCB SPE Cartridge, 250 mg, 3 mL; ANPEL, Shanghai, China, www.anpel.com.cn/cnw), filtrated (SCAA-104, 0.22-μm pore size; ANPEL, Shanghai, China, http://www.anpel.com.cn/) and used for liquid chromatography-mass spectrometry analysis (Lang *et al.* 2021).

### High-performance liquid chromatography conditions

The sample extracts were analysed using an liquid chromatography electrospray ionisation tandem mass spectrometry (LC-ESI-MS)/MS system (high-performance liquid chromatography [HPLC], Shim-pack UFLC SHIMADZU CBM30A system, www.shimadzu.com.cn/; MS, Applied Biosystems 4500 QTrap, www.appliedbiosystems.com.cn/). The analytical conditions were as follows: HPLC column, Waters ACQUITY UPLC HSS T3 C18 (1.8 µm, 2.1 mm * 100 mm); solvent system, water (0.04 % acetic acid):acetonitrile (0.04 % acetic acid); gradient program, 100:0 V/V for 0 min, 5:95 V/V for 11.0 min, 5:95 V/V for 12.0 min, 95:5 V/V for 12.1 min, 95:5 V/V for 15.0 min; flow rate, 0.40 mL min^−1^; temperature, 40 °C; injection volume, 5 μL. The effluent was alternatively connected to an ESI-triple quadrupole-linear ion trap (QTrap)-MS.

### Qualitative and quantitative analysis of metabolites

Qualitative analysis of primary and secondary metabolites was carried out by comparing the accurate precursor ions (Q1), product ion (Q3) values, retention times (RT) and fragmentation patterns with those obtained by injecting standards under the same conditions when standards were available (Sigma-Aldrich, USA, http://www.sigmaaldrich.com/united-states.html). When standards were not available, qualitative analysis was conducted using the self-compiled database MWDB (MetWare Biological Science and Technology Co., Ltd, Wuhan, China) and publicly available metabolite databases. Repeated signals of K^+^, Na^+^, NH^4+^ and other high-molecular-weight substances were eliminated during the identification. Quantitative analysis of metabolites was performed in maximum reference match (MRM) mode. The characteristic ions of each metabolite were screened using a QQQ mass spectrometer to obtain signal strengths. Integration and correction of chromatographic peaks were performed using MultiQuant version 3.0.2 (AB SCIEX, Concord, Ontario, Canada). The corresponding relative metabolite contents were represented by chromatographic peak area integrals.

### ESI-QTrap-MS/MS

LIT and triple quadrupole (QQQ) scans were acquired on a triple quadrupole-linear ion trap mass spectrometer (QTrap), API 6500 QTrap LC/MS/MS System, equipped with an ESI Turbo Ion-Spray interface, operating in positive ion mode and controlled by Analyst 1.6 software (AB Sciex). The ESI source operation parameters were as follows: ion source, turbo spray; source temperature 500 °C; ion spray voltage (IS) 5500 V; ion source gas I (GSI), gas II (GSII), curtain gas (CUR) were set at 55, 60 and 25.0 psi, respectively; the collision gas (CAD) value was high. Instrument tuning and mass calibration were performed with 10 and 100 μmol L^−1^ polypropylene glycol solution in QQQ and LIT modes, respectively. QQQ scans were acquired as MRM experiments with collision gas (nitrogen) set to 5 psi. declustering potential (DP) and collision energy (CE) values for individual MRM transitions were obtained via further DP and CE optimization. A specific set of MRM transitions was monitored for each period based on the metabolites eluted within this period.

## Results

### Purple pigment accumulation in FQ-36 curds

**Figure 1.**
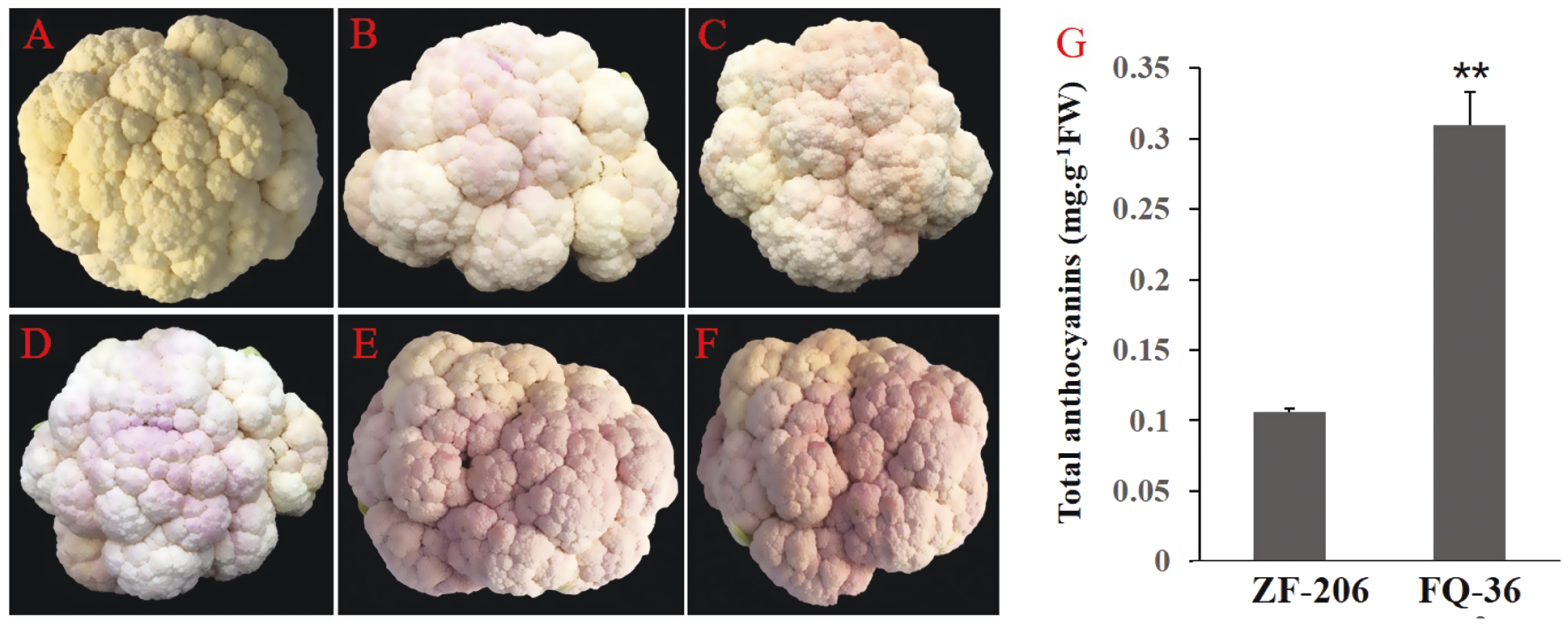
Phenotype of different cauliflower curds and anthocyanins abundance analysis. (A) Light orange curds of ZF-206; (B–D) the curds with light purple part and white part of FQ-36 cauliflower in cold weather; (E and F) different plants curd colour of purple FQ-36 cauliflower in cold weather; Scale bar of A–F: 5 cm; (G) total anthocyanins content of ZF-206 and FQ-36 purple cauliflower curd. For total anthocyanins content analysis, both ZF-206 and FQ-36 are with three replicates, the error bars represent difference between three repetitions of anthocyanins content analysis, and the ** means statistically significant with *t*-test *P*-value < 0.01.

At the seedling stage, ZF-206 plants had green hypocotyls, cotyledons and young leaves, whereas purple pigments strongly accumulated in the hypocotyls of FQ-36 plants **[see****Supporting Information—Fig. S1****]**. In the winter, purple pigments gradually and strongly accumulated in FQ-36 curds with decreasing temperature, whereas but no such pigments were detected in ZF-206 curds (Fig. 1A–F). We measured the total anthocyanin contents of fully purple FQ-36 curds and white ZF-206 curds, revealing significantly higher anthocyanin contents in purplish curds (Fig. 1B; **see****Supporting Information—Table S1**).

### Identification of anthocyanins by LC-ESI-MS/MS-based metabolic profiling

**Figure 2.**
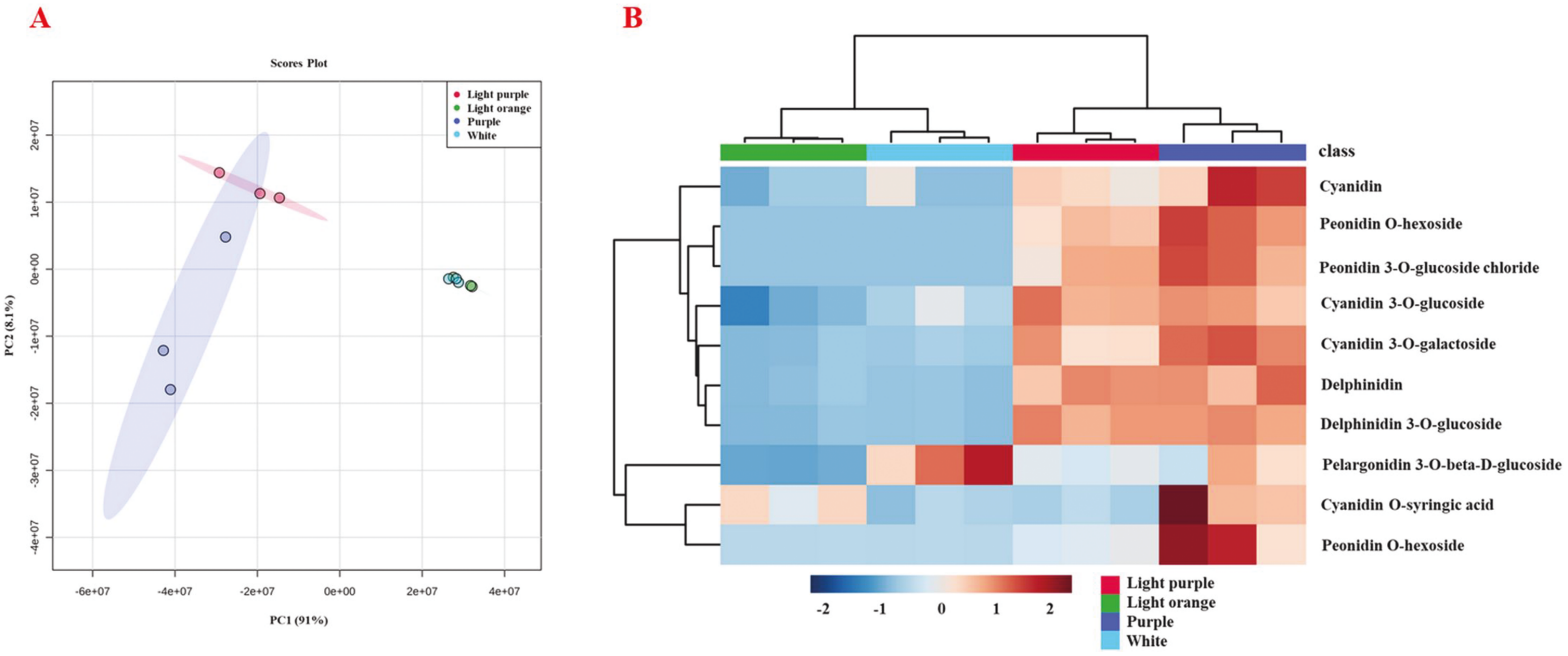
Cluster heat map of anthocyanin metabolites and content. (A) PCA analysis of the metabolites for the four groups; (B) heat map of the metabolites for the four groups.

To further analyse the anthocyanin compositions in curds of different colours, we conducted LC-ESI-MS/MS-based metabolite profiling to assess the variation in secondary metabolite contents in the four samples. In the winter of 2018, we collected white curds of ZF-206 and fully purple curds of FQ-36. During the development of purplish FQ-36 curds with decreasing temperature, the white parts and light purple parts of the same curds were also collected. All four samples were subjected to metabolic profiling analysis with three metabolic repeats. This analysis identified 10 anthocyanidins, including cyanidin, peonidin, delphinidin, pelargonidin and petunidin (Table 1; **see****Supporting Information—Table S4**). Principal component analysis (PCA) and clustering analysis based on these metabolite measurements demonstrated that light orange curds and white curds contained very similar metabolite compositions, whereas light purple curds contained similar metabolites to those of fully purple curds, which showed much more variation in different duplicate samples (Fig. 2A and B).

**Table 1.** Identified anthocyanins in cauliflower curds by LC-ESI-MS/MS-based metabolic profiling.

Groups	Compounds	Molecular weight	Ionization model	MS/MS (m/z)	Relative content			
					Light orange (ZF-206)	White (FQ-36)	Middle purple (FQ-36)	Purple (FQ-36)
Cyanidin	Cyanidin	287.24	Protonated	287	165 899 ± 146 958a	71 757 ± 45 179b	444 656 ± 54 243c	749 863 ± 188 297d
	Cyanidin 3-*O*-galactoside	448.1006	[M]+	287	4 773 800 ± 1 064 066a	2 130 233 ± 1 467 483b	30 204 333 ± 6 717 896c	44 342 000 ± 2 748 037d
	Cyanidin *O*-syringic acid	466.1	[M-H]−	287	81 970 ± 46 611a	370 060 ± 84 247b	99 152 ± 29 297	700 423 ± 274 380
	Cyanidin 3-*O*-glucoside	448.3	[M-H]−	287	67 127 ± 15 800a	24 269 ± 14 300b	147 747 ± 12 963	1 423 567 ± 12 498
	Peonidin *O*-hexoside	463.123	Protonated	303	9 ± 0a	9 ± 0a	78 933 ± 10 134b	124 626 ± 14 681c
	Peonidin 3-*O*-glucoside chloride	498.0929	[M-Cl]+	465/303	9 ± 0a	9 ± 0a	73 565 ± 16 987b	103 804 ± 16 256c
Delphinidin	Delphinidin	303.24	Protonated	303	43 533.3 ± 13 835.0a	34 538 ± 29 242a	771 987 ± 92 828b	819 027 ± 113 109c
	Delphinidin 3-*O*-glucoside	465.1	Protonated	303	3 906 100 ± 658 432a	2 434 300 ± 1 617 643a	47 641 000 ± 3 511 659b	47 948 000 ± 2 122 402c
	Petunidin 3-*O*-glucoside	479	Protonated	465/303	90 418 ± 21 439a	98 023 ± 20 009a	5 693 133 ± 1 243 859b	27 860 333 ± 11 681 141c
Pelargonidin	Pelargonidin 3-*O*-beta-D-glucoside	433.1	Protonated	271	1 450 333 ± 332 286a	212 980 ± 15 423b	722 360 ± 43 808c	944 073 ± 286 225d

Compared to the light orange curds of ZF-206, the relative levels of all identified anthocyanins were significantly higher in light purple curds and fully purple curds of FQ-36, except for pelargonidin 3-*O*-beta-D-glucoside (Table 1). The content of pelargonidin 3-*O*-beta-D-glucoside was significantly higher in light orange ZF-206 curds than in the light purple parts of FQ-36 curds. Compared to the white parts of FQ-36 curds, the levels of all anthocyanins were higher in the light purple parts of these curds and in fully purple curds, except for cyanidin *O*-syringic acid, which was present at lower levels in the light purple parts of FQ-36 curds (Table 1). The highest of five anthocyanins also significantly differed between light orange ZF-206 curds and white FQ-36 curds, including cyanidin *O*-syringic acid, cyanidin 3-*O*-glucoside, pelargonidin 3-*O*-beta-D-glucoside, cyanidin and cyanidin *O*-galactoside. Interestingly, except for cyanidin *O*-syringic acid, whose levels were highest in the white parts of FQ-36 curds, the levels of all other anthocyanins were highest in the light orange curds of ZF-206 (Table 1).

### RNA-seq and DEGs analyses of white versus purplish cauliflower curds

**Figure 3.**
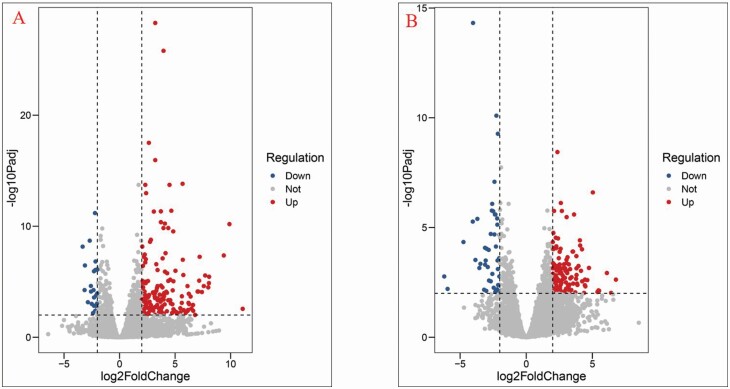
Volcano plot of the DEGs between cauliflower curds with different phenotype. (A) DEGs between light orange curds and purple curds; (B) DEGs between middle-white and middle-purple groups. Criteria: *P*adj < 0.01 and log_2_FoldChange > 2.

To identify the genes involved in purplish curd development, we performed comparative RNA-seq using the same samples used for metabolic analysis, including light orange ZF-206 curds, fully purple FQ-36 curds and white and light purple parts of the same FQ-36 curds. All four samples were subjected to transcriptome sequencing with three biological repeats. In total, we obtained 24 million reads, which were aligned to the *B. oleracea* reference genome (TO1000). The uniquely mapped reads, with mapping rates ranging from 88.23 to 89.54 %, were used for further analysis **[see****Supporting Information—Table S2****]**. Differentially expressed genes between samples were identified using DEseq2 with the following criteria: *P*adj < 0.01 and log_2_FoldChange > 2. We identified 189 DEGs between purplish FQ-36 curds and ZF-206 curds, including 166 genes that were upregulated and 23 genes that were downregulated in purplish curds (Fig. 3A). We also identified 170 DEGs between the white parts and light purple parts of FQ-36 curds, including 126 that were upregulated and 44 that were downregulated in the light purple parts of curds (Fig. 3B).

To compare the expression patterns of anthocyanin-related genes between white and purplish curds, we identified 94 homologous genes in *B. oleracea* var. TO1000 by BLAST analysis using the protein sequences of 45 anthocyanin biosynthetic enzymes and regulators from *Arabidopsis* as queries. Three transcription factor genes were differentially expressed between ZF-206 curds and the white parts of FQ-36 curds, including *LBD37*, *LBD39* and *EGL3*. Four anthocyanin biosynthetic genes were differentially expressed between the white and light purplish parts of FQ-36 curds, including *DFR*, *UGT79B1*, *PAL1* and *F3′H*. Three (*DFR*, *UGT79B1*, *ANS*) and five genes (*DFR*, *UGT79B1*, *ANS*, *PAL4* and *EGL3*) were significantly downregulated in ZF-206 curds and the white parts of FQ-36 curds, respectively, compared to purple FQ-36 curds. Many more DEGs were identified between the white parts of FQ-36 curds and fully purple curds, including *ANS*, *MYBL2*, *4CL2*, *4CL3*, *PAL2*, *FLS5*, *EGL3*, *DFR*, *TT8*, *UGT79B1* and *TT19*. However, only one gene (*PAL1*) was differentially expressed between the light purplish parts of FQ-36 curds and fully purple FQ-36 curds **[see****Supporting Information—Table S3****]**.

**Figure 4.**
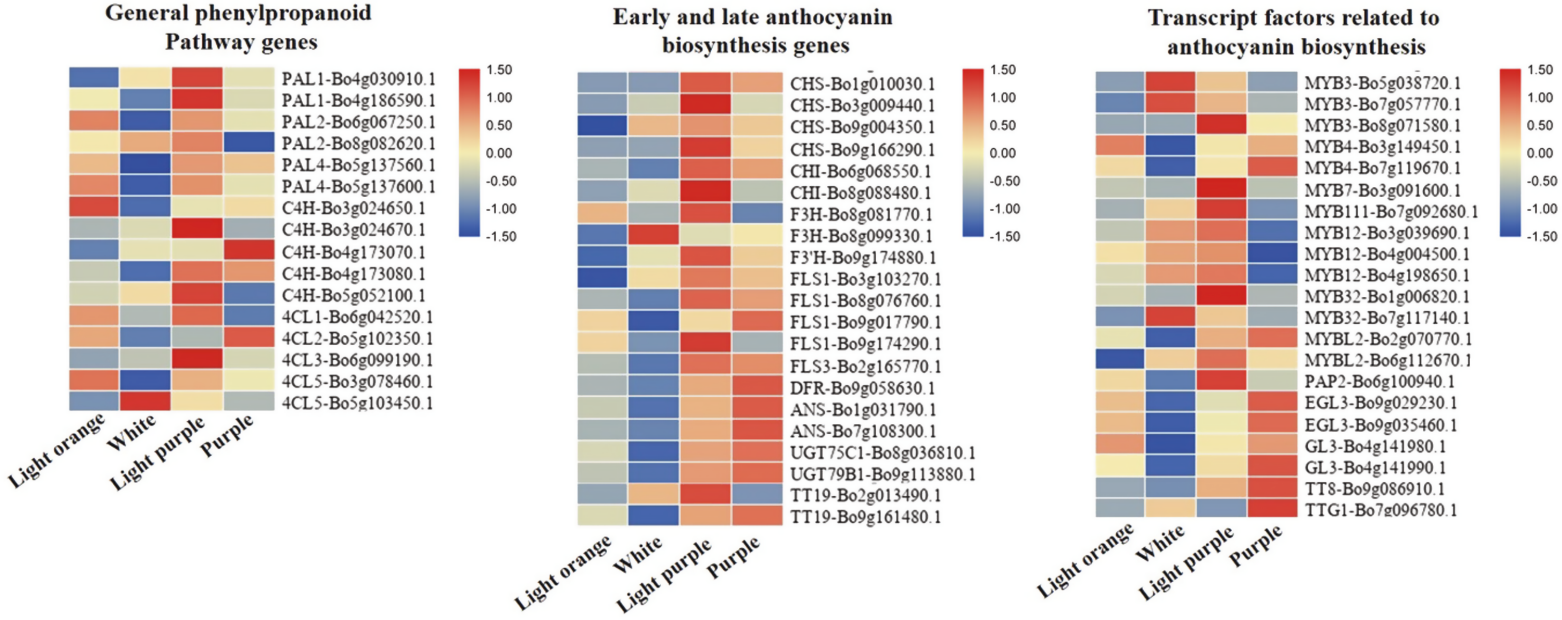
Heatmap of the expression of the genes in anthocyanin biosynthetic pathway. The expression level of general phenylpropanoid pathway genes is higher in light purple group, while the early and late anthocyanin biosynthesis genes and transcript factors were highly expressed in light purple group and purple group.

**Figure 5.**
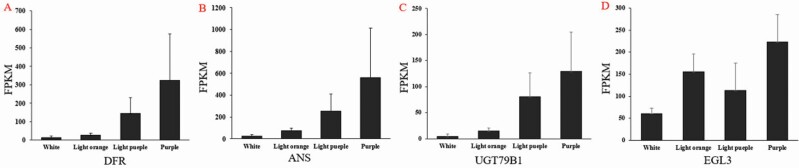
The expression of *DFR*, *ANS*, *UGT79B1*, *EGL3* in cauliflower curds with increased colour of different cauliflower curds, each sample with three replicates. *DFR*, *ANS*, *UGT79B1* are LBGs of anthocyanin biosynthesis pathway, and *EGL3* is one of the key transcript factors of bHLH family that regulate anthocyanin biosynthesis, they directly regulate the biosynthesis of anthocyanins.

We generated heat maps of all expressed anthocyanin biosynthetic genes in the four samples (Fig. 4). Most of the anthocyanin biosynthetic and regulatory genes were expressed at higher levels in light purplish curds and in the fully purple parts of FQ-36 curds compared to light orange ZF-206 curds and the white parts of FQ-36 curds; these results are consistent with the phenotypes of these curds. Compared to the light purplish parts of curds, phenylpropanoid pathway genes, EBGs and most genes encoding transcription factors controlling EBG expression were expressed at lower levels in the fully purple parts of FQ-36 curds than in the other samples, whereas LBGs and genes encoding MBW complex components, including *TTG1*, *TT8*, *GL3* and *EGL3*, were highly expressed in these samples. Interestingly, most genes in the general phenylpropanoid pathway, LBGs and related transcription factor genes were expressed at higher levels in light orange ZF-206 curds, but most EBGs were expressed at lower levels in these curds compared to the white parts of FQ-36 curds. For example, the expression of four genes (*DFR*, *ANS*, *UGT79B1*, *EGL3*) gradually increased with increasing accumulation of purplish pigments in the white parts, light purplish parts and fully purple parts of FQ-36 curds (Fig. 5). However, these genes were expressed at higher levels in light orange ZF-206 curds than in the white parts of FQ-36 curds, which is not consistent with the phenotypes of the curds. Meanwhile, although *PAP2* was not one of the DEGs identified in each comparison **[see****Supporting Information—Table S2****]** due to the poor repeatability in one of the three duplicates, its average expression level was significantly higher in light purple curds than in any other samples. The qRT-PCR analysis of *PAP2*, *DFR*, *ANS* indicated that they are significantly upregulated expressed in purplish FQ-36 curds, while it is hardly expressed in light orange ZF-206 curds (Fig. 7).

**Figure 6.**
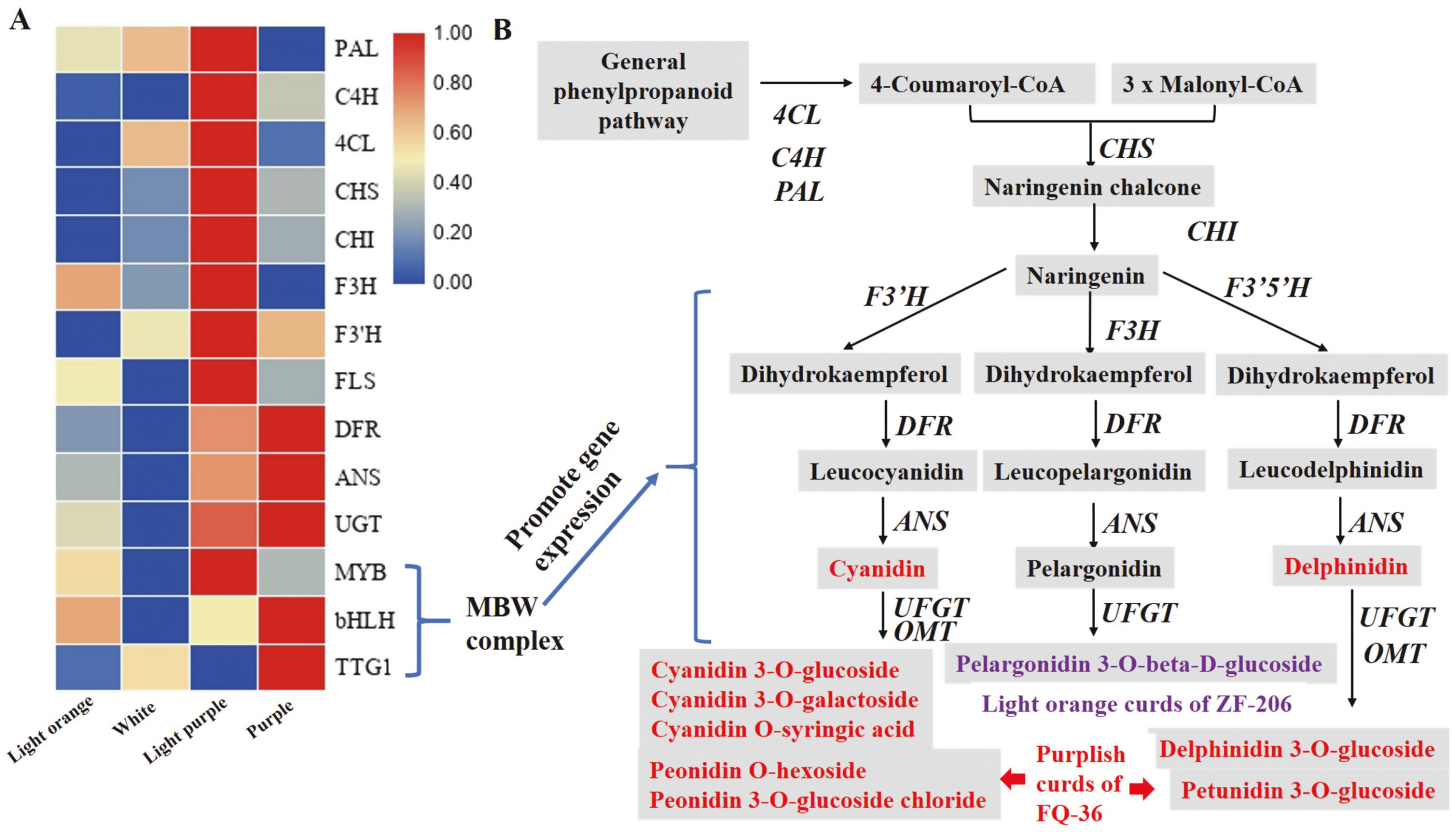
The expression of structural genes and transcription factors in anthocyanin metabolism pathway. (A) The expression of structural genes and transcription factors in anthocyanin metabolism pathway of each sample; (B) synthetic pathway and type of anthocyanins in FQ-36.

**Figure 7.**
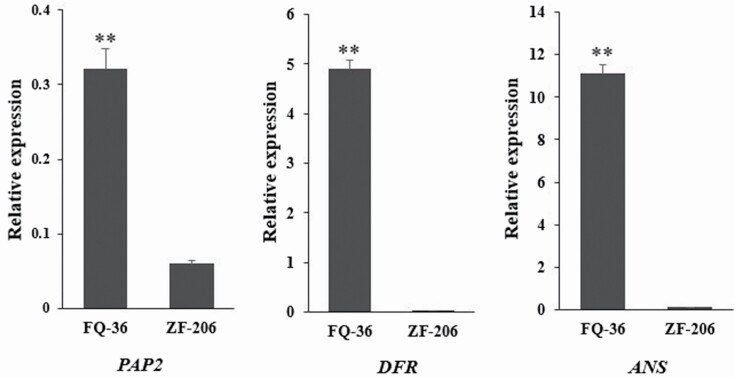
qRT-PCR analysis of *PAP2*, *DFR* and *ANS* relative expression in FQ-36 and ZF-206. For qRT-PCR analysis, both ZF-206 and FQ-36 are with three replicates, the error bars represent difference between three repetitions of anthocyanins content analysis compared to the reference Bnaactin3 gene, and the ** means statistically significant with *t*-test *P*-value < 0.01.

## Discussion

### Purplish curds form due to anthocyanin accumulation

Four enzymes in the anthocyanin biosynthesis pathway, CHI, F3H, DFR and ANS, are required to synthesize the coloured anthocyanidin pelargonidin. However, the hydroxyl groups on the B ring are determined by two cytochrome P450s, flavonoid 3′-hydroxylase (F3′H, classified as CYP75B) and flavonoid 3′5′-hydroxylase (F3′5′H; mainly CYP75A); these proteins lead to the generation of cyanidin and delphinidin, respectively (Tanaka and Brugliera 2013). Cyanin can be further methylated to form peonidin, and delphinidin can be methylated to form petunidin or malvidin. Depending on many factors, pelargonidin generally appears orange to red, cyanidin appears red-purple and delphinidin appears red-purple to blue (Zhang *et al.* 2014a). Anthocyanin production and accumulation have been reported in purple cauliflower, such as the variety ‘Graffiti’, which results from a natural mutation (Chiu *et al.* 2010), and ‘Sicilian purple’, which is botanically intermediate between broccoli and cauliflower (Branca *et al.* 2017).

Analysis by HPLC alone or HPLC combined with quadrupole tandem mass spectrometry (HPLC-MS/MS) revealed that cyanidin is predominant in these purple cauliflower curds (Lo Scalzo *et al.* 2008; Singh *et al.* 2020). Cyanidin is also the predominant anthocyanin in various other *Brassica* species, such as red cabbage (*B. oleracea* var. *gongylodes*) (Wu and Prior 2005), purple kohlrabi (*B. oleracea* var. *gongylodes*) (Zhang *et al.* 2015), purple bok choy (*B. rapa* var. *chinensis*) (Zhang *et al.* 2014b), *B. napus* (Goswami *et al.* 2018; Yin *et al.* 2019) and purple tumorous stem mustard (*B. juncea* var. *tumida*) (Xie *et al.* 2014). Similarly, in the current study, two anthocynidin and six anthocyanin molecules were detected in cauliflower curds, six of which are cyanidins or derived peonidins (Table 1). Interestingly, two delphinidin or derived petunidin and one type of pelargonidin were also detected in purplish and light orange curds. In particular, the content of pelargonidin 3-*O*-beta-D-glucoside was significantly higher in light orange curds than in curds of other colours. These results indicate that the formation of purplish curds results from the accumulation of more cyanidins and delphinidins in the surfaces of the curds, whereas light orange curd formation results from the strong accumulation of cyanidins and pelargonidins.

The sequences of *CYP75A* (*F3′5′H*) subfamily members are not represented in the *Arabidopsis* genome, which explains the absence of delphinidin in this species (Bak *et al.* 2011). The same is true for *B. rapa* (Guo *et al.* 2014) and perhaps for *B. oleracea* and *B. napus* as well, since their genomes do not contain *CYP75A* sequences. However, delphinidin has been detected in *B. rapa* (purple bok choy) (Zhang *et al.* 2014b) and purple heading Chinese cabbage (He *et al.* 2016), *B. oleracea* (red cabbage) (Lin *et al.* 2008), *B. napus* (Fu *et al.* 2018; Goswami *et al.* 2018; Yin *et al.* 2019) and cauliflower (the present study). Thus, it would be interesting to investigate whether *B. rapa*, *B. oleracea* and *B. napus* contain isoenzymes that share the same catalytic activity as F3′5′H. For example, some Asteraceae species have re-acquired F3′5′H activity from their *F3′H* gene by convergent evolution (Seitz *et al.* 2006).

### Potential genes for the formation of purplish curds

Gene expression analysis based on RNA-seq revealed significant differences between light orange ZF-206 curds and both light purple and fully purple FQ-36 curds, which showed significantly higher expression of the late anthocyanin biosynthesis genes *ANS*, *DFR* and *UGT79B1* and two transcription factor genes, *PAP2* and *EGL3*. In particular, the expression levels of four genes (*DFR*, *ANS*, *UGT79B1*, *EGL3*) increased gradually from the white to light purplish to fully purple parts of FQ-36 curds. Late biosynthesis genes are regulated by MBW complexes composed of WD40, bHLH and MYB transcription factors. In *Arabidopsis*, bHLH transcription factors include TT8, GL3 and EGL3, which play redundant roles in complex formation and the transcriptional activation of LBGs (Nesi *et al.* 2000). GL3 and EGL3 contribute equally to the activation of *F3′H*, but EGL3 appears to play a more predominant role in the activation of DFR and ANS genes, which function in *Arabidopsis* seedling pigmentation (Gonzalez *et al.* 2008). These observations are consistent with the finding that anthocyanin accumulated at high levels in light purple and purple curds but at very low levels in white curds. Surprisingly, two genes encoding MYBL2, a negative regulator of anthocyanin accumulation (Shi and Xie 2014), were also highly expressed in light purple curds and purple curds, respectively. Highly expressed *MYBL2* homologues were also detected in purple leaves of *B. rapa* (Mushtaq *et al.* 2016), but the reason for this is currently unknown.

However, because *EGL3* is also expressed at significantly higher levels in light orange ZF-206 curds than in white and even light purple FQ-36 curds, this is unlikely the key factor responsible for the very low purple pigment accumulation on the surfaces of ZF-206 curds. Moreover, although the relative cyanidin and delphinidin contents were very low in ZF-206 curds, these curds had significantly higher pelargonidin 3-*O*-beta-D-glucoside contents (Table 1; Fig. 2). These results are consistent with the low activity of F3′H in ZF-206 curds; this enzyme catalyses the production of cyanidin and delphinidin.

Because the expression of *F3′H* is regulated by the MBW complex, and considering the relatively high expression level of *EGL3* in ZF-206 curds, the low expression level of *PAP2* (also known as *BoMYB2*, Bo6g100940) may the reason for the low activity of F3′H in ZF-206 curds. Although *PAP2* was expressed at a low level in purplish curds, it was expressed at a significantly higher level in light purple curds (Fig. 6), qRT-PCR analysis of *PAP2* indicated that it is significantly upregulated expressed in purplish FQ-36 curds (Fig. 7). Previously studies indicated that purple curd colour in cauliflower is due to an insertion of a Harbinger transposon in the promoter region of *BoMYB2* (Chiu *et al.* 2010, 2012). The mutations in the promoter region of *BoMYB2* are also responsible for purple colour formation in the leaves and stems of different types of *B. oleracea* variants, including purple cabbage, purple kale and purple kohlrabi (Yan *et al.* 2019). BoMYB2 is involved in MYB–bHLH–WD40 complex formation by directly interacting with BobHLH1. The expression of *BoMYB2* in *Arabidopsis* significantly upregulated a subset of anthocyanin structural genes, including *F3′H*, *DFR* and *ANS* (Chiu and Li 2012). Altogether, these observations indicate that the strong expression of *PAP2* is the key reason for the purple pigment accumulation in the surfaces of white FQ-36 curds, although additional experiments are needed to confirm this finding.

## Conclusion

Phenotypic, metabolic and transcriptome sequencing analysis indicated that purplish curds in cauliflower result from the accumulation of cyanidin and delphinidin in the curd surface. This is likely due to the strong expression of *PAP2*, encoding a key component of the MBW complex, in purplish curds.

## Supporting Information

The following additional information is available in the online version of this article—

Figure **S**1. Seedling phenotype of ZF-206 and FQ-36. In the seedling stage, the hypocotyl of FQ-36 has obvious purple production, but ZF-206 does not.

Table **S**1. Total anthocyanins content of white and purple cauliflower curds.

Table S2. The unique mapped reads number and mapping rate of each sample.

Table **S**3. Differentially expressed anthocyanin-related genes between each sample.

Table S4. Raw data of metabolic analysis of anthocyanin (Table 1).

plac001_suppl_Supplementary_Material

plac001_suppl_Supplementary_Table_S4

## Data Availability

All materials and related data in this study are available upon request.

## Notes

**Phenome, Genome & Environment. Chief Editor**: Colleen Doherty
