# Supplementary material for: Metabolic and RNA sequencing analysis of cauliflower curds with different types of pigmentation
Source: AoB Plants. 2022 Feb 5;14(2):plac001. doi: 10.1093/aobpla/plac001 (PMC8994856; doi:10.1093/aobpla/plac001)
Supplement: plac001_suppl_Supplementary_Material [file plac001_suppl_supplementary_material.pdf]

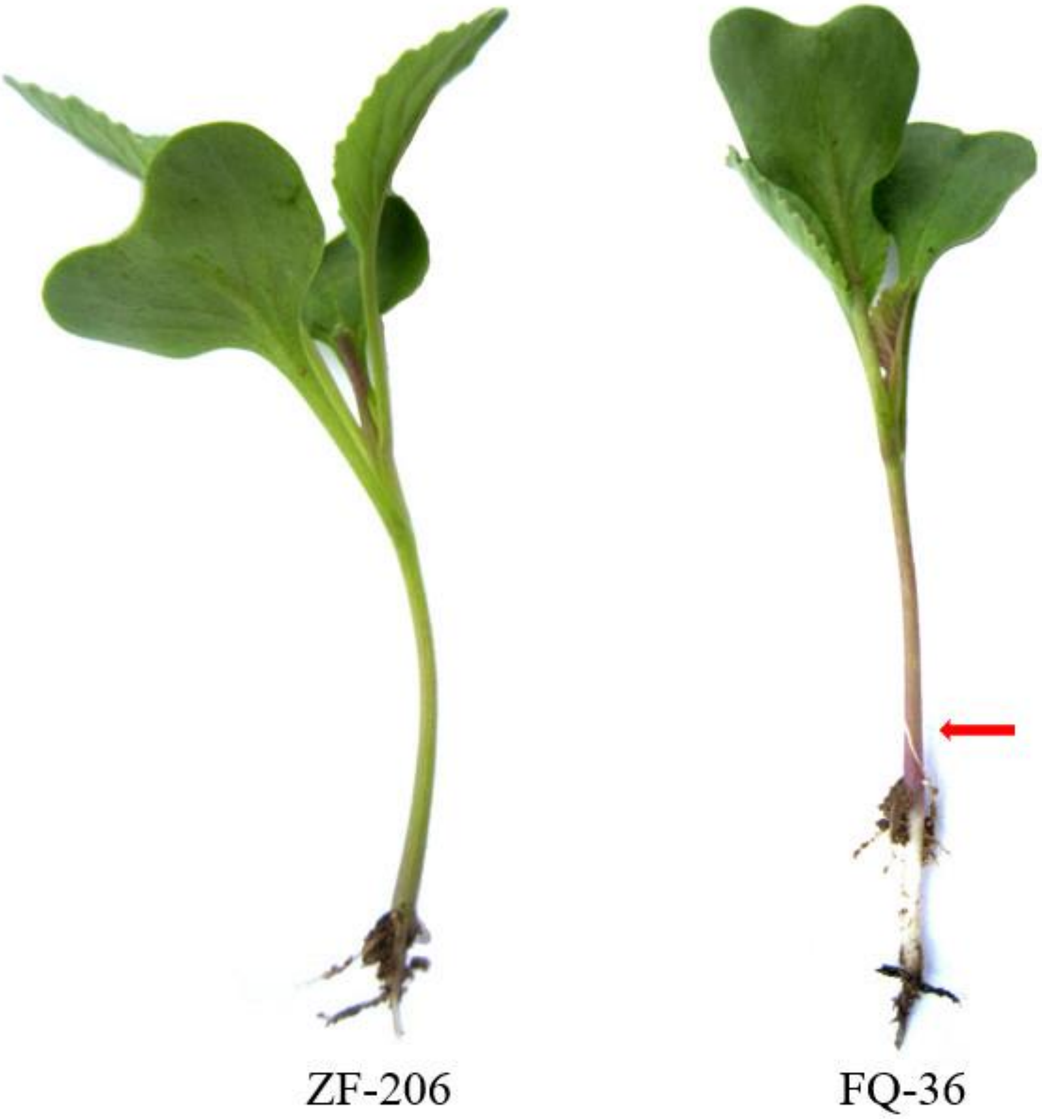

Supplementary figure 1 Seedling phenotype of ZF-206 and FQ-36. In the seedling stage, the hypocotyl of FQ-36 has obvious purple production, but ZF-206 does not.

Supplementary Table 1. Total anthocyanins content of white and purple cauliflower curds

Phenotype	mg.g-1FW
White	0.1043294
	0.1043294
	0.1087824
Purple	0.3289618
	0.3162529
	0.2834956

Supplementary Table 2. The unique mapped reads number and mapping rate of each sample

phenotype	name	reads number	mapping rate
White	No_1	24155888	89.34%
	No_2	25478457	89.20%
	No_3	24435453	87.92%
Purple	No_4	24570589	87.95%
	No_5	23140211	89.41%
	No_6	27029374	89.05%
Light orange	No_7	24755390	88.93%
	No_8	26249411	89.15%
	No_9	24714793	88.65%
Light purple	No_10	24844869	89.54%
	No_11	24522819	88.23%
	No_12	23725796	88.81%

Supplementary Table 3. Differentially expressed anthocyanin-related genes between each sample

	white	Middle white	Middle purple	Purple
White	****	Bo9g035460 (EGL3) Bo1g003550 (LBD39) Bo7g063630 (LBD37)	Bo4g030910 (PAL1) Bo9g058630 (DFR) Bo9g113880 (UGT79B1) Bo9g174880 (F3'H)	Bo1g031790 (ANS) Bo9g058630 (DFR) Bo9g113880 (UGT79B1)
Light orange		*****	Bo1g031790 (ANS) Bo5g137560 (PAL4) Bo9g035460 (EGL3) Bo9g058630 (DFR) Bo9g113880 (UGT79B1)	Bo1g031790 (ANS) Bo2g070770 (MYBL2) Bo5g102350 (4CL2) Bo6g027640 (4CL3) Bo8g082620 (PAL2) Bo9g017790 (FLS5) Bo9g035460 (EGL3) Bo9g058630 (DFR) Bo9g086910 (TT8) Bo9g113880 (UGT79B1) Bo9g161480 (TT19)
Light purple			*****	Bo8g082620 (PAL2)
Purple				*****
